# Transforming quality use of medicines in Saudi Arabia: physician perspectives on electronic prescribing systems

**DOI:** 10.3389/fdgth.2026.1812544

**Published:** 2026-07-29

**Authors:** Fahad Aldhafeeri, Andrew Wilson, Shaun Larkin

**Affiliations:** 1 Leeder Centre for Health Policy, Economics and Data, Faculty of Medicine and Health, University of Sydney, Sydney, NSW, Australia; 2 Hafr Albatin Health Cluster, Ministry of Health, Hafr Albatin, Saudi Arabia

**Keywords:** effectiveness, efficiency, electronic prescribing, physicians, quality use of medicines

## Abstract

**Background:**

Electronic prescribing (EP) systems are now a core component of hospital healthcare in Saudi Arabia (SA), aiming to enhance patient safety and streamline complex clinical workflows. There is, however, limited evidence on how physicians in large government hospitals that use multiple prescribing platforms view these systems in daily practice, as most prior research has focused on primary care or on single academic centres. This study aimed to evaluate physicians' perspectives on the effectiveness and efficiency of EP systems across multiple government hospitals in a major Saudi city and to identify key advantages and obstacles associated with their use.

**Methods:**

A cross-sectional survey, adapted from validated international tools, was conducted in March 2025 among physicians in seven government hospitals. The questionnaire comprised four sections: demographics, efficiency (18 items), effectiveness (10 items), and open-ended questions on perceived advantages and challenges. Data was analysed using descriptive statistics to summarise responses, Pearson's chi-squared test to examine associations between variables, and thematic analysis for free-text comments.

**Results:**

A total of 167 physicians completed the survey; most were male (82.6%), and nearly half were general practitioners (46.1%). Overall satisfaction with EP systems was high, with respondents reporting perceived improvements in workflow efficiency (79.0%), patient safety (91.0%), error reduction (91.0%), and guideline adherence (86.8%). Mean scores on a five-point scale were 4.30 (SD = 0.82) for efficiency and 4.59 (SD = 0.71) for effectiveness. Efficiency scores were significantly associated with physician gender (*p* < 0.05) and specialty (*p* < 0.01). Physicians perceived EP as associated with reduced administrative burden, faster error correction, and smoother coordination with pharmacists, while commonly reported challenges included poor internet reliability, system downtime, incomplete medication lists, and limited training.

**Conclusion:**

Physicians in Saudi government hospitals generally view EP systems as beneficial, helping to streamline workflows, enhance patient safety, and support evidence-based practice. Nevertheless, technical issues, training gaps, and workflow disruptions indicate that these systems are not yet operating at full potential. Addressing these challenges through targeted training, infrastructure upgrades, and improved interprofessional communication is essential to maximise the benefits of digital prescribing and align with international best practices.

## Background

Electronic prescribing (e-prescribing or EP) systems have become a core component of contemporary medication management. They are widely promoted as a strategy to advance the quality use of medicines (QUM). EP enables the digital transmission of prescriptions between prescribers and dispensers, replacing handwritten orders with standardised, legible, and structured electronic orders. International evidence indicates that EP is associated with significant reductions in prescribing errors, improvements in adherence to guidelines and workflows, and cost savings, although the magnitude of benefit varies across settings and system designs (1–3).

In Saudi Arabia (SA), EP adoption forms part of broader national efforts to serve different healthcare levels and strengthen digital health infrastructure. The Ministry of Health's Wasfaty platform electronically links primary care centres and community pharmacies and is increasingly used for outpatient prescriptions in hospitals, aiming to improve access, continuity of supply, and prescription tracking (4). However, Wasfaty has been reported to have limited integration with electronic health records and to implement only a subset of best-practice computerised provider order entry (CPOE) features (5), while users have highlighted challenges such as insufficient training and high prescription volumes (6). In parallel, hospital information systems such as Careware and OASIS provide more comprehensive EP functionality within government hospitals, including formulary management, allergy and interaction checks, and integration with other clinical modules (7, 8). Despite these advances, studies documented usability problems, technical barriers, intermittent downtime, incomplete medication lists, workflow disruptions and alert fatigue as recurring obstacles that can affect user satisfaction and medication safety (9–12). The successful adoption of EP systems significantly depends on user acceptability, contentment, and engagement, with favourable perceptions linked to increased efficiency, diminished medication mistakes, and improved care coordination (13, 14). Consequently, understanding how frontline clinicians experience EP in routine practice remains critical for realising its potential to enhance QUM rather than digitise existing processes.

Existing research on EP in SA has focused mainly on patient satisfaction (15–17) and community pharmacy experiences (6, 18–20) with the Wasfaty service, with only limited work exploring physicians' perspectives, often restricted to primary care or single academic centres using a single EP platform. Only two studies were found that evaluated physicians' perceptions about EP; one study focused exclusively on primary healthcare centres (PHCs) (21), and the other study assessed a single academic medical centre (22), and both focused on a specific EP program. As a result, there is little multi-site evidence on how physicians working in large government hospitals, and using multiple EP systems, perceive the efficiency and effectiveness of EP in their daily practice. The specific advantages and challenges they identify, and how these relate to QUM goals, also remain underexplored.

This study addresses these gaps by surveying physicians across seven government hospitals in a major Saudi city to assess their satisfaction with EP systems, perceived effects on efficiency and effectiveness, and key implementation barriers and enablers, to inform targeted improvements to hospital-based digital prescribing.

## Methods

### Study design

A cross-sectional survey was conducted in March 2025 among physicians working in seven government hospitals in Hafr Albatin, a major city in SA that routinely use EP systems. All physicians working in the seven government hospitals and using EP systems were eligible, and the participation was voluntary. Hospital administration and department heads were responsible for distributing the survey link via institutional email and internal messaging groups. This study is part of a PhD project evaluating the QUM policies and programs in SA.

### Sample

According to the Health Cluster (a regional organisation of healthcare providers that aims to deliver healthcare to a defined population within a specific geographic area in SA), 800 physicians work at the chosen hospitals, which comprise all government hospitals in a single major Saudi city. All of these hospitals use EP systems, and including every hospital in the city was intended to capture the full range of EP use in this setting and to generate findings representative of the local MOH hospital system. According to the Raosoft equation (23), the required sample size for a 5% margin of error at a 95% confidence level was 260. Of the 260 surveys distributed, 167 were completed, yielding a response rate of 64.2%. Participation was limited mainly by workload and scheduling constraints reported informally by department coordinators.

### The survey questionnaire

The survey instrument, adapted from Raeesi et al. (24) by removing items specific to insurance coverage and reimbursement and adding items on specific local EP platforms. The survey was reviewed by clinical informatics experts after adaptation. Internal consistency reliability was assessed for the efficiency (18 items) and effectiveness (10 items) scales using Cronbach's alpha. The efficiency scale showed excellent internal consistency (Cronbach's alpha = 0.96), and the effectiveness scale showed close results (Cronbach's alpha = 0.97). It consists of four sections:

1. **Demographics:** Age, gender, years of experience, and practice setting.
2. **Efficiency** (18 items): Questions on time savings, workflow integration, administrative burden, and cost-effectiveness.
3. **Effectiveness** (10 items): Questions on patient safety, error reduction, guideline adherence, and continuity of care.
4. Open-ended questions about the positive and negative aspects of using the electronic prescribing system.

All questions were rated on a 5-point Likert scale (1 = strongly disagree, 5 = strongly agree). The survey was distributed electronically, and participation was voluntary and anonymous.

### Data analysis

Data for demographics, effectiveness, and efficiency items were analysed using descriptive statistics, including means, standard deviations, frequencies, and percentages, to summarise participant demographics and responses to survey items rated on a 5-point Likert scale. Pearson's chi-squared, independent-samples t-tests, and one-way analysis of variance (ANOVA) test were used to compare variables, with *p* < 0.05 considered statistically significant. In addition, multivariate analysis was done to examine independent predictors of overall efficiency and effectiveness scores. Demographic variables were entered as covariates, and standard regression assumptions were checked and met. For the open-ended responses, thematic analysis was conducted by two researchers, who first thoroughly read the responses to gain a comprehensive understanding, a process called familiarisation. Then, the data were coded by labelling and identifying recurring responses, focusing on the benefits and challenges of EP. The discussion and comparison between the identified odes were conducted to develop a shared coding framework. Finally, the codes were grouped into themes and subthemes. Results were presented in tables for clarity.

### Ethical Approval

This study was approved by the National Committee of Bioethics, SA (H-05-FT-083). Written informed consent was not obtained, and completion of the questionnaire was taken as implied consent, as approved by the ethics committee.

## Results

### Physician characteristics

The survey included 167 participants, predominantly male (82.6%). Most of the participants were under 55 years old. Only 8.4% were aged 25–34 years, and 6% were over 55 years. Regarding their professional background, 46.1% identified as general practitioners, 43.7% as specialists, and 10.2% as consultants. In terms of departmental affiliation, 37.7% worked in medical services, 35.5% in emergency departments (ED), and 27% in surgical services. Physician characteristics are summarised in Table 1.

**Table 1:** Demographics of the physicians who participated in the study.

Variable	Category	Frequency	Percentage
Gender	Male	138	82.6%
	Female	29	17.4%
Age group	25–35 years	14	8.4%
	35–45 years	71	42.5%
	45–55 years	76	45.5%
	>55 years	6	6.0%
Specialisation	General Practitioner	77	46.1%
	Specialist	73	43.7%
	Consultant	17	10.2%
Computer skills	Elementary	28	16.8%
	Intermediate	108	64.7%
	Advanced	31	18.6%
Electronic prescribing system	Wasfaty	42	25.1%
	Careware	92	55.1%
	OASIS	33	19.8%

### Information system experience

When assessing their computing skills, most rated themselves as intermediate (64.7%), 18.6% considered themselves advanced, and 16.8% as elementary. The predominant prescribing systems participants had experienced were Careware (55.1%), Wasfaty (25.1%), and OASIS (19.8%).

### Efficiency

Respondents reported agreement with multiple items relating to the efficiency of electronic prescribing. The majority agreed with items stating that the system improves workflow efficiency (79.0%), saves time with patients (79.0%), reduces patient waiting times (80.2%), and decreases the time required for medication prescribing compared to traditional methods (80.8%). Similarly, most respondents agreed that EP facilitates faster error correction (83.8%), reduces treatment costs (82.0%), and reduces the number of prescriptions per patient (84.4%).

Respondents also agreed with items relating to physician workload (79.6%), illegible handwriting (91.0%), and patient visits to medical centres (85.0%). Agreement was also high for items related to increasing overall task efficiency (82.0%), making tasks easier for physicians (79.6%), and enabling medication and procedure registration in fewer steps (83.8%). Neutral responses ranged from 6.0% to 20.0% across items, and negative responses did not exceed 14% for any item. Item-level responses are presented in Table 2.

**Table 2:** An itemised view of how respondents rated each aspect of efficiency and effectiveness in electronic prescribing.

Category	Dimension/Item	Mean (SD)	Strongly Disagree + Somewhat Disagree	Neither Agree nor Disagree	Strongly Agree + Somewhat Agree
1. Efficiency	Dimension of Time				
	More time is allocated to patients	4.04 (1.23)	22 (13.2%)	27 (16.2%)	118 (71.1%)
	Patient waiting time is reduced	3.99 (1.25)	27 (16.2%)	23 (13.8%)	117 (70.1%)
	Less time is spent on prescriptions compared to traditional methods	4.06 (1.27)	24 (14.4%)	17 (10.2%)	126 (75.5%)
	Mistakes can be corrected in the shortest time	4.39 (1.05)	13 (7.8%)	10 (6%)	144 (86.3%)
	Patients' time is saved when receiving medications	4.29 (1.03)	12 (7.2%)	24 (14.4%)	131 (78.5%)
	Physicians' time is saved	4.11 (1.18)	19 (11.4%)	27 (16.2%)	121 (72.5%)
	Task performance is sped up	4.29 (1.02)	15 (9.0%)	16 (9.6%)	136 (81.5%)
	1.1. Dimension of Cost				
	Treatment costs are generally reduced	4.28 (1.01)	7 (3.8%)	38 (22.8%)	122 (73.1%)
	Savings occur in medication prescribing and use	4.35 (1.03)	10 (6.0%)	19 (11.4%)	138 (82.7%)
	The number of prescriptions per patient is reduced	4.49 (0.91)	9 (5.4%)	10 (6%)	148 (88.7%)
	Organisational resources are saved through medication registration	4.41 (0.97)	8 (4.8%)	23 (13.8%)	136 (81.5%)
	1.2. Other Efficiency Related Items				
	The system is easy to use and requires little mental effort	4.35 (1.00)	14 (8.4%)	10 (6%)	143 (85.6%)
	Physicians' workload is reduced	4.11 (1.12)	17 (10.2%)	30 (18%)	120 (71.9%)
	Prescribing issues (e.g., illegible handwriting) are reduced	4.80 (0.63)	3 (1.8%)	2 (1.2%)	162 (97.0%)
	Patients' visits to medical centres are reduced	4.41 (0.97)	9 (5.4%)	17 (10.2%)	141 (84.5%)
	Greater efficiency is achieved in performing tasks	4.48 (0.85)	6 (3.6%)	16 (9.6%)	145 (86.9%)
	Task performance for physicians is easier	4.33 (1.02)	12 (7.2)	18 (10.8%)	137 (82.1%)
	Medication and procedure registration require fewer steps	4.37 (1.03)	14 (8.4%)	10 (6%)	143 (85.7%)
2. Effectiveness	2.1. Dimensions of Patient Safety				
	Patient safety is increased	4.69 (0.77)	5 (3.0%)	5 (3%)	157 (94.0%)
	Medication prescribing is improved according to guidelines	4.63 (0.77)	5 (3.0%)	6 (3.6%)	156 (93.5%)
	Medical errors are fewer	4.66 (0.78)	5 (3.0%)	5 (3%)	157 (94.0%)
	Care in providing services is increased	4.55 (0.81)	6 (3.6%)	7 (4.2%)	154 (92.3%)
	System alerts contribute to better medication prescribing	4.64 (0.87)	8 (4.8%)	5 (3%)	154 (92.2%)
	Access to patients' medication history improves prescribing	4.69 (0.73)	6 (3.6%)	3 (1.8%)	158 (94.6%)
	2.2. Other Effectiveness-Related Items				
	Medication registration occurs during patient visits	4.56 (0.86)	7 (4.2%)	8 (4.8%)	152 (91.1%)
	All intended medications are registered	4.58 (0.74)	9 (5.4%)	4 (2.4%)	154 (92.3%)
	Greater control over daily medication prescribing is facilitated	4.62 (0.80)	6 (3.6%)	7 (4.2%)	154 (92.2%)
	General effectiveness in prescribing is increased	4.44 (0.94)	8 (4.8%)	21 (12.6%)	138 (82.7%)

Efficiency-related aspects had a mean score of 4.30 (SD = 0.82) on the five-point scale. Data analysis showed a significant relationship between efficiency and gender (*P* < 0.05), with females scoring significantly lower than males. In addition, a highly significant relationship between efficiency and specialisation (*P* < 0.01) was observed, with consultants scoring significantly lower than other specialisations. Table Three represents the association between efficiency/effectiveness and physician characteristics.

### Effectiveness

Perceptions of the system's effectiveness were similarly favourable (Table 2). The vast majority of physicians agreed that electronic prescribing increases patient safety (91.0%), improves adherence to prescribing guidelines (86.8%), and reduces medical errors (91.0%). High agreement was also observed for items related to increasing care in service delivery (86.2%), the value of system alerts in improving medication prescribing (89.8%), and the benefit of access to patients' medication history for better prescribing decisions (91.0%).

Physicians also endorsed the system's role in facilitating better information exchange between physicians and pharmacies (85.6%), eliminating some work demands on physicians (82.6%), and improving medication registration during patient visits (85.6%). Most agreed that all intended medications are registered (88.6%), that the system enables greater control over daily medication prescribing (85.6%), and that it increases physicians' overall effectiveness in prescribing medications (84.5%). Neutral responses for effectiveness items ranged from 6.0% to 18.0%, while negative responses remained below 12% across all items.

Effectiveness-related aspects of the EP system received a mean score of 4.59 (SD = 0.71), indicating strong user agreement with improvements in patient safety and medication management. There was no significant relationship between effectiveness and any of the respondents' characteristics (Table 3).

**Table 3:** The mean score of participants in each category and the univariate analysis according to the demographic data.

Demographic category	Efficiency		Effectiveness	
	Mean (SD)	*P*-value	Mean (SD)	*P*-value
Gender				
Male	4.37 (0.79)	0.023*	4.63 (0.67)	0.139
Female	3.99 (0.89)		4.41 (0.86)	
Age group				
25–35	3.96 (0.82)	0.11	4.36 (0.76)	0.59
35–45	4.30 (0.83)		4.59 (0.75)	
45–55	4.41 (0.76)		4.64 (0.68)	
>55	3.81 (1.18)		4.58 (0.60)	
Specialization				
General Practitioner	4.39 (0.80)	0.004*	4.62 (0.78)	0.263
Specialist	4.37 (0.79)		4.63 (0.66)	
Consultant	3.69 (0.81)		4.32 (0.56)	
Computer skills				
Elementary	4.55 (0.74)	0.185	4.69 (0.79)	0.719
Intermediate	4.28 (0.83)		4.58 (0.73)	
Advanced	4.17 (0.83)		4.55 (0.59)	
Electronic prescribing system				
WASFATY	4.20 (0.84)	0.591	4.48 (0.83)	0.236
CAREWEARE	4.31 (0.86)		4.58 (0.73)	
OASIS	4.40 (0.67)		4.76 (0.44)	

Comparisons of overall efficiency and effectiveness scores across the three electronic prescribing systems using one-way ANOVA showed no statistically significant or practically important differences between systems. However, there was a statistically significant association between overall efficiency and effectiveness scores. In the multivariable model, efficiency was statistically significant and explained 10% of the variance [R² = 0.10, adjusted R² = 0.06; F(7,159) = 2.59, *p* = 0.015], indicating that these characteristics together accounted for about 10% of the variability in perceived efficiency. In contrast, effectiveness was not statistically significant and explained very little variance (R² = 0.05, adjusted R² = 0.01). Overall, the multivariable analyses showed low explained variance for efficiency and effectiveness. In addition, there was no evidence of a combined impact of demographic factors (age, gender, and specialisation) on users' perceptions.

### Advantages and challenges of EP systems

Analysis of physicians' responses to open-ended questions about the benefits and challenges of EP has identified several important themes. When asked what they thought were the benefits of the EP systems, they were most frequently reported as faster (*n* = 101), more accurate (*n* = 65), and more efficient (*n* = 44). Many respondents reported that the system alerts and access to comprehensive medication histories enhanced safety (*n* = 80) and reduced medical errors (*n* = 112), particularly those caused by illegible handwriting. It was also highlighted that the system improved coordination between physicians and pharmacists (*n* = 32), simplified record-keeping (*n* = 21), and reduced administrative workload and resource consumption (*n* = 44). Convenience and ease of use (*n* = 19) were emphasised. Some physicians noted that the system saves time for both physicians and patients and supports better compliance with regulatory requirements.

**Figure 1:**
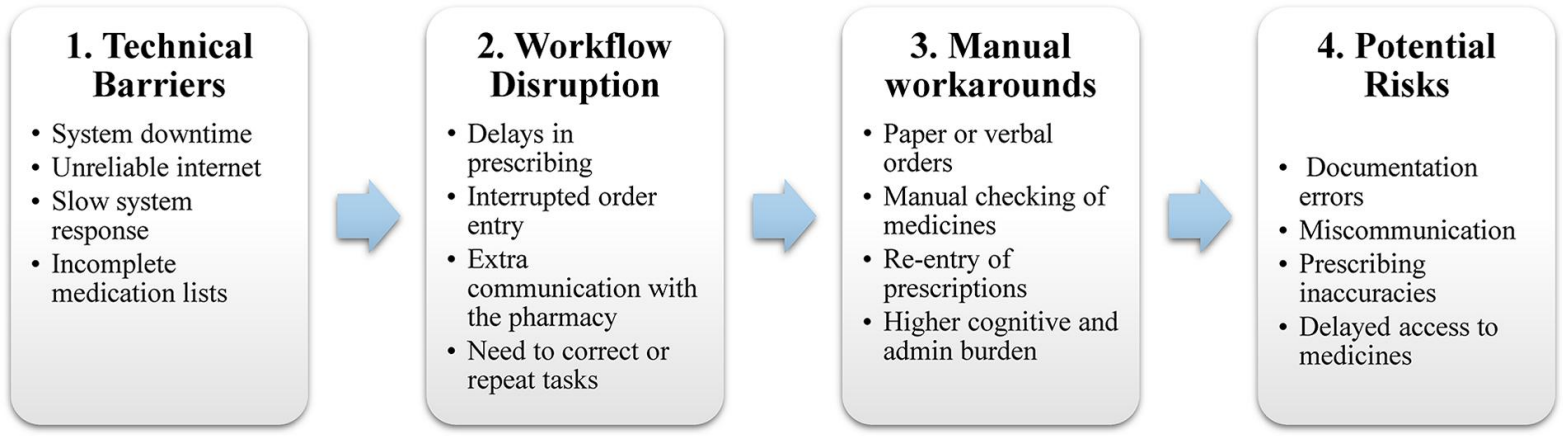
A pathway from technical problems to workflow disruption and possible medication safety risks.

However, participants reported unreliable internet connections (*n* = 69), system downtime (*n* = 73), and incomplete medication lists (*n* = 55) as challenges associated with the use of EP. Figure 1 illustrates the pathway from EP system downtime and technical issues to clinical workflow disruption and potential medication safety risks. Training requirements were also cited by users as a widespread issue (*n* = 48), with some staff struggling to transition from paper to electronic systems and finding the process challenging and time-consuming. There were concerns about data security and privacy, as well as fears of data loss or system failures (*n* = 23). Some users believe the system is harder to use than traditional methods, particularly when they encounter some technical issues. They also underlined the need for continuous improvements in infrastructure, user support, and system design to harness the advantages of electronic prescribing fully. Key themes and frequencies from open-ended responses are listed in Table 4.

**Table 4:** Key themes identified from the response of physicians' perspectives about the advantages and challenges of electronic prescribing systems.

Benefits		
Theme	Subtheme	Example from Respondents
Operational Efficiency	Time Savings & Workflow Streamlining	“The system saves time for both doctors and patients and reduces waiting times.”
	Administrative Burden Reduction	“It simplifies record-keeping and lessens the administrative workload.”
	Cost-Effectiveness	“Leads to cost savings through reduced treatment and resource use.”
Safety and Accuracy	Reduction in Medical Errors	“With EP, we have fewer issues related to illegible handwriting and reduced medical errors.”
	Enhanced Decision Support	“The system alerts help improve medication prescribing and patient safety.”
	Access to Medication History	“Access to patients' medication history allows for better prescribing decisions.”
Coordination and Compliance	Physician-Pharmacist Communication	“Improving coordination between physicians and pharmacists.”
	Regulatory Adherence	“It supports better compliance with legal and regulatory requirements.”
Convenience and Accessibility	Ease of Use	“The system is convenient and easy to use.”
	Patient Access to Medications	“Medications are more readily available and accessible for patients, especially those with chronic conditions.”
Challenges		
Theme	Subtheme	Example from Respondents
Technical Limitations	System Downtime & Internet Issues	“Unreliable internet connections and system downtime are significant barriers.”
	Incomplete Medication Lists	“The software sometimes lacks adequate medication options, making prescribing difficult.”
Adaptation and Training Barriers	Insufficient Training	“Some staff struggle to adjust to the transition from paper to electronic systems due to a lack of training.”
	Resistance to Change	“Many physicians are used to paper prescriptions and are reluctant to accept the changes.”
	Workflow Disruption	“The transition is time-consuming and sometimes disrupts our usual workflow.”
Infrastructure and Support	Data Security and Privacy Concerns	“There are fears of data loss or breaches in patient confidentiality.”
	Lack of Technical Support	“Inadequate infrastructure and support negatively impact the system's adoption.”
	Need for Continuous Optimisation	“Continuous enhancements in infrastructure and system design are needed.”

## Discussion

### Statement of principal findings

The findings of this study reflect physicians' perceptions of EP rather than direct clinical outcomes. The study shows that most Saudi physicians consider EP systems useful and report perceived improvements in efficiency and effectiveness. Respondents reported that EP is associated, in their experience, with streamlining workflow, reducing patients' waiting times, and speeding up error correction. They also noted benefits such as better coordination with pharmacists, easier record-keeping, and improved regulatory compliance. Despite this overall high satisfaction, physicians reported challenges including system downtime, unreliable internet connections, and incomplete medication lists.

### Interpretation within the context of wider literature

Two studies from Iran used the same validated survey instrument developed by Raeesi et al., which was also adapted for the current study (24, 25). Those studies found only average ratings for EP efficiency and effectiveness, with scores below 60% of the maximum. In contrast, our study reveals substantially higher satisfaction and perceived benefits among hospital physicians in SA. However, cross-country differences usually reflect cultural variation in response styles and expectations for digital systems, but they can also reflect true differences in system performance. Physicians in this study emphasised the importance of adequate training and ongoing professional development to maintain efficiency and effectiveness. Other research from SA has shown that tailored EP modifications can reduce prescribing errors by about 50% (8), and several studies underline the need for physicians and pharmacists to be actively involved in the design and implementation phases to make sure that systems meet the needs of clinical practice (9, 26).

In addition, the findings of this study are consistent with the Unified Theory of Acceptance and Use of Technology (UTAUT), which highlights key determinants of technology acceptance, moderated by user characteristics such as gender, age, and experience, including performance expectancy, effort expectancy, social influence, and facilitating conditions. The high efficiency and effectiveness scores reflect strong performance expectancy, while the generally positive views on ease of use and workload indicate favourable effort expectancy. At the same time, challenges such as limited training, system downtime and unstable internet access highlight weaknesses in facilitating conditions, which the UTAUT framework considers essential for sustained system use. The small but meaningful differences in efficiency across gender and specialty are also consistent with UTAUT's view that user characteristics control how these determinants influence acceptance and use. This underscores the need for role-sensitive implementation strategies rather than a uniform approach (27).

#### Stakeholders' disparities in SA

In contrast to the physicians in this survey, pharmacists in SA have reported moderate to low satisfaction with the use of electronic prescribing systems and reported significant operational barriers like increased workload (6, 18), medication shortages (17, 19, 20), and communication gaps with prescribers (17, 18, 20) as critical challenges, leading to low satisfaction. This interprofessional gap underscores the need for systematic reforms, improved communication, and shared training to optimise the EP system. Similarly, the EP systems in SA have received moderate to significant dissatisfaction from patients with medication shortages and poor physician-pharmacist communication (15–17). While drug shortages are primarily logistical or supply chain issues independent of the EP platform itself, some dissatisfaction appears linked to outdated or incomplete medication formularies within the EP system. Patients may think that problems arise when doctors prescribe items that are not available but remain on the system's medicine list. This indicates that there have to be real-time updates to the formulary and better interaction between EP systems and pharmacy inventory databases to ensure that the list of accessible medications appropriately shows stock levels at dispensing sites. Table 5 presents the key findings from studies evaluating the perspectives of different stakeholders on EP systems in SA.

**Table 5:** Key findings of studies evaluating electronic prescribing (EP) systems in Saudi Arabia (SA).

Study & Year	Region	Design	E-prescribing system	Sample	Key Benefits	Primary Barriers
Alghamdi et al. (2023) (18)	Al Baha, Najran, Jazan (Southern province)	Cross-sectional survey	Automated pharmacy systems (APS)	403 pharmacists	- Potential for improved care - Enhanced medication safety	- Limited APS adoption (50% never used e-prescriptions) - Staff shortages - Lack of training (85%) - Poor physician collaboration
Alsahali et al. (2023) (19)	Qassim (Middle Province)	Descriptive survey	Wasfaty	124 pharmacists	- Reduced dispensing errors (68.6%) - Reduced ambiguities (75.8%) - Reduced forgeries (70.2%)	- Medication unavailability (88.7%) - Prescriber errors (83.9%) - Technical issues (46%) - Increased workload (81.5%)
Khardali et al. (2022) (20)	Jazan (Southern province)	Qualitative interviews	Wasfaty	9 pharmacists	- Reduced dispensing errors - Facilitated medication refills - Reduced medication waste	Medication unavailability - Inability to dispense alternatives - Lack of pharmacist-prescriber communication - Inadequate facilities
Alharthi (2024) (6)	Makkah (western province)	Qualitative interviews	Wasfaty	15 pharmacists	- Improved prescription legibility - Reduced medication errors - Streamlined dispensing	- Often low (especially in chronic patients) Frequent medication shortages - Inability to provide alternatives - Lack of direct prescriber communication - Increased administrative burden
Almaghaslah et al. (2022) (17)	National	Patient satisfaction survey	Wasfaty	400 Patients	- Good pharmacist availability - Clear medication instructions	- Medication shortages -limited availability of a suitable area for counselling to maintain privacy
Tobaiqy et al. (2023) (16)	Jeddah (Eastern Province)	Cross-sectional survey	Wasfaty	217 Patients	- Initial system registration satisfaction	- Medication access issues - Communication gaps
Rasheed et al. (2024) (15)	National	Cross-sectional survey	Wasfaty	718 Patients	-Easier renewal and refills of medications -Accessibility to pharmacies	-Medication availability issues -limited area suitable for counselling in community pharmacies

#### Gender and implementation nuances

Multivariable analysis showed that female physicians reported slightly lower efficiency scores than their counterparts. In contrast, perceptions of effectiveness remained uniformly high and were not independently associated with any measured demographic variables. This study sample had a substantial gender imbalance, with the majority of participants being male. This reflects the male dominance in the current healthcare workforce in SA (approximately 65% of physicians are male) (28), and the relatively recent entry of women into full clinical practice (29). Both male and female physicians acknowledged their role in enhancing patient safety and medication management. However, male physicians rated the EP system higher than their female counterparts in both efficiency and effectiveness, although the difference in the latter was not statistically significant. This implies that gender may affect how physicians perceive the efficiency of EP and suggests that females experience more workflow disruptions due to system design. The gap in ratings may be attributed to the limited participation of women in decision-making and administrative roles, and to the presence of systems designed to favour male-dominated workflows, as reported previously by women in the healthcare sector in SA (30). The underrepresentation of women in decision-making roles and the possibility that systems have been designed around male-dominated workflows. Although gender disparities in digital health satisfaction have been examined in larger contexts, no research has specifically identified male–female differences in satisfaction with EP systems. This underscores the need for further investigation into the impact of gender dynamics on the adoption of these systems, especially in healthcare systems undergoing workforce transitions, such as SA.

#### Specialty and seniority trends

In this study, consultants reported lower efficiency scores than junior physicians even after adjustment for other measured characteristics, although the multivariable model explained only about 10% of the variance in efficiency. For example, a study found that more experienced physicians, notably consultants and specialists in endocrinology, neurology, and infectious diseases, were more likely to rate their EHR and EP experiences poorly. They reported that using EP systems was associated with usability frustrations and an increased documentation burden (31). Another study that found low overall satisfaction with EP systems among over half of the physicians showed that dissatisfaction is often higher among senior clinicians, who face more challenges adapting to new digital workflows and cite technical and design issues as critical barriers (32). These senior clinicians frequently manage more complex cases and have high expectations for system performance and personalisation, making them more sensitive to inefficiencies, inflexible workflows, and the absence of advanced features. In addition, long-established routines based on paper or older systems can make the shift to digital platforms harder for consultants. Previous studies also suggest that training and support are less often tailored to senior users, which may contribute to their lower satisfaction. These observations indicate a need for personalisation, specialty-specific enhancements, and role-sensitive training so that EP works effectively for clinicians at all levels. However, because potential confounders such as case complexity, workload, and primary EP system were not adjusted for, these explanations for consultants' lower satisfaction remain tentative rather than confirmed.

### Strengths and limitations

This study used a validated tool to evaluate the perspectives of physicians working in large government hospitals in a rapidly digitalising health system. It has the advantage of a multi-site design, which helps capture diverse views, and of integrating qualitative and quantitative data. However, this study is at risk of non-response bias, as physicians with stronger opinions about EP may have been more likely to participate than those with heavier workloads, who may have been under-represented. Also, the study depends on self-reported perception and does not measure objective indicators such as error rates, time-motion data, or clinical outcomes. Also, a significant limitation of this study is the gender disparity among participants, with the sample predominantly male. This reflects the current composition of the healthcare workforce in SA, but it may have affected the findings on user satisfaction, particularly in how male and female physicians perceive the efficiency and effectiveness of EP systems. Female physicians, who remain underrepresented, may have experiences that this study does not fully capture. Another limitation is that despite participants reporting use of three different EP platforms (Wasfaty, Careware, and OASIS), the study did not compare satisfaction or usability across these systems. Future research should aim for more gender balance and investigate system-specific experiences to inform targeted improvements.

### Implications for policy, practice and research

To address these shared and context-specific challenges, future systems should emphasise continuous digital training for both professions, infrastructure upgrades, and stronger interprofessional communication channels, ensuring active involvement of physicians and pharmacists in designing and implementing digital health solutionsIn addition, ongoing evaluation and refinement of system design, aligned with real-world clinician workflows, are crucial for enhancing safety and performance. It is also recommended to upgrade the EP platforms to fully comply with international best practices, particularly in system integration and clinical decision support. For example, upgrading EP systems to include real-time Application Programming Interface (API) integration between EP systems, the National Formulary, and pharmacy inventory databases would help ensure that available stock is accurately reflected at the point of prescribing and dispensing (33).

## Conclusion

Physicians who participated in this study emphasised that system efficiency and patient safety were among the most important perceived benefits. Still, they also identified technical glitches, training gaps, and workflow disruptions as key barriers undermining optimal EP adoption.

## Data Availability

The raw data supporting the conclusions of this article will be made available by the authors, without undue reservation.
